# Chronic pain diagnoses and opioid dispensings among insured individuals with serious mental illness

**DOI:** 10.1186/s12888-020-2456-1

**Published:** 2020-01-31

**Authors:** Ashli Owen-Smith, Christine Stewart, Musu M. Sesay, Sheryl M. Strasser, Bobbi Jo Yarborough, Brian Ahmedani, Lisa R. Miller-Matero, Stephen C. Waring, Irina V. Haller, Beth E. Waitzfelder, Stacy A. Sterling, Cynthia I. Campbell, Rulin C. Hechter, John E. Zeber, Laurel A. Copeland, Jeffrey F. Scherrer, Rebecca Rossom, Greg Simon

**Affiliations:** 1 Health Policy and Behavioral Sciences, School of Public Health, Georgia State University, Urban Life Building, 140 Decatur Street, Suite 434, Atlanta, GA 30303 USA; 2 Center for Research and Evaluation, Kaiser Permanente Georgia, Atlanta, USA; 3 Health Research Institute, Kaiser Permanente Washington, Seattle, USA; 4 Center for Health Research, Kaiser Permanente Northwest, Portland, USA; 5 Center for Health Policy and Health Services Research, Henry Ford Health System, Detroit, USA; 6 Depart Behavioral Health Services, Henry Ford Health System, Detroit, USA; 7 Essentia Institute of Rural Health, Duluth, USA; 8 Center for Health Research, Kaiser Permanente Hawaii, Honolulu, USA; 9 Division of Research, Kaiser Permanente Northern California, Oakland, USA; 10 Department of Research and Evaluation, Kaiser Permanente Southern California, Pasadena, USA; 11 School of Public Health & Health Sciences, University of Massachusetts Amherst, Amherst, USA; 12 A Central Western Massachusetts Healthcare, Leeds, MA USA; 13 Department of Family and Community Medicine, Saint Louis University School of Medicine, Saint Louis, USA; 14 Health Partners Institute, Minneapolis, USA

**Keywords:** Chronic non-cancer pain, Opioids, Serious mental illness

## Abstract

**Background:**

Individuals with major depressive disorder (MDD) and bipolar disorder (BD) have particularly high rates of chronic non-cancer pain (CNCP) and are also more likely to receive prescription opioids for their pain. However, there have been no known studies published to date that have examined opioid treatment patterns among individuals with schizophrenia.

**Methods:**

Using electronic medical record data across 13 Mental Health Research Network sites, individuals with diagnoses of MDD (*N* = 65,750), BD (*N* = 38,117) or schizophrenia or schizoaffective disorder (*N* = 12,916) were identified and matched on age, sex and Medicare status to controls with no documented mental illness. CNCP diagnoses and prescription opioid medication dispensings were extracted for the matched samples. Multivariate analyses were conducted to evaluate (1) the odds of receiving a pain-related diagnosis and (2) the odds of receiving opioids, by separate mental illness diagnosis category compared with matched controls, controlling for age, sex, Medicare status, race/ethnicity, income, medical comorbidities, healthcare utilization and chronic pain diagnoses.

**Results:**

Multivariable models indicated that having a MDD (OR = 1.90; 95% CI = 1.85–1.95) or BD (OR = 1.71; 95% CI = 1.66–1.77) diagnosis was associated with increased odds of a CNCP diagnosis after controlling for age, sex, race, income, medical comorbidities and healthcare utilization. By contrast, having a schizophrenia diagnosis was associated with decreased odds of receiving a chronic pain diagnosis (OR = 0.86; 95% CI = 0.82–0.90). Having a MDD (OR = 2.59; 95% CI = 2.44–2.75) or BD (OR = 2.12; 95% CI = 1.97–2.28) diagnosis was associated with increased odds of receiving chronic opioid medications, even after controlling for age, sex, race, income, medical comorbidities, healthcare utilization and chronic pain diagnosis; having a schizophrenia diagnosis was not associated with receiving chronic opioid medications.

**Conclusions:**

Individuals with serious mental illness, who are most at risk for developing opioid-related problems, continue to be prescribed opioids more often than their peers without mental illness. Mental health clinicians may be particularly well-suited to lead pain assessment and management efforts for these patients. Future research is needed to evaluate the effectiveness of involving mental health clinicians in these efforts.

## Background

Chronic non-cancer pain (CNCP) affects an estimated 25.3 million Americans [1] at a cost of $600 billion [2]. The use of long-term opioid therapy as a treatment for CNCP has quadrupled in the last 15 years [3–5] despite little empirical evidence that opioids are effective for treating CNCP long-term [6, 7] and has instead resulted in dramatic increases in opioid abuse and overdose deaths [8, 9]. In order to more effectively address this epidemic, we need to better understand which populations are most burdened by CNCP and which populations are at the greatest risk of opioid use/abuse in order to guide both clinical and policy-related decisions.

Evidence suggests that individuals with mental illness may be one population with particularly high rates of CNCP and may also be more likely to receive prescription opioids for their pain. Several studies have reported that individuals with depression and bipolar disorder, for example, have more frequent pain complaints, higher pain intensity and more pain chronicity and are also significantly more likely to receive long-term opioids, at a higher daily dose, and with greater days supplied compared with patients without mental illness [10–16]. By contrast, evidence suggests that CNCP is less prevalent among individuals with schizophrenia compared to individuals without mental illness [17]; to our knowledge, there have been no studies published to date that have examined opioid treatment patterns specifically among individuals with schizophrenia compared to controls.

This gap in the literature, in addition to other methodological limitations inherent in many prior studies – including small sample sizes [13, 18] and limited generalizability (e.g., examining only military veterans) [11, 15, 19] – prompted the present study. Specifically, we investigated (1) whether individuals with major depressive disorder (MDD), bipolar disorder (BD) and schizophrenia are more or less likely to receive a chronic pain diagnosis compared to individuals with no psychiatric diagnoses and (2) whether individuals with MDD, BD and schizophrenia are more or less likely to receive chronic prescription opioid medications compared to individuals with no psychiatric diagnoses using data from health care systems in the Mental Health Research Network (MHRN) that are representative of a large, geographically and racially/ethnically diverse population across the U.S.

## Methods

### Data source

The MHRN consists of 13 research centers located within large integrated health care delivery systems, serving over 12.5 million individuals across 15 states; most of these delivery systems also have affiliated health insurance plans. All MHRN sites maintain a Virtual Data Warehouse consisting of electronic health record (EHR) and insurance claim data for all enrolled members or patients. Data on encounters, pharmacy fills, diagnoses, laboratory tests and demographics are organized using standardized definitions across sites and are quality checked locally [20].

The current study involved 10 MHRN systems. These sites were 6 Kaiser Permanente sites (Georgia, Washington, Northwest, Hawaii, Northern California, Southern California), Henry Ford Health System, Essentia Health, Baylor Scott and White Healthcare and Health Partners. Institutional Review Boards at each site approved the study protocol for this project.

### Study population

Individuals were included if they met the following criteria: adults aged 18–70 years (as of January 1, 2016) with a diagnosis of MDD (ICD-9296.2–296.39/ICD-10 F32-F33.9), BD (ICD-9296.0x, 296.1x, 29.40–296.89/ICD-10 F30-F31.9) or schizophrenia including schizoaffective disorder (ICD-9295.x/ICD-10 F20.x, F25.x) documented at least two times by mental healthcare provider in 2015 or 2016 (cases had to “start” 2016, the 12-month study period, with a diagnosis so at least 1 diagnosis had to occur in 2015). Patients who had diagnoses in more than 1 of these categories were categorized hierarchically: schizophrenia>BD > MDD. For example a patient with schizophrenia and MDD would be classified in the schizophrenia group and a patient with only MDD would be classified in the MDD group. This is an approach used in prior studies that have similarly employed a hierarchy of non-overlapping categories [21, 22]. Eligible individuals had to have continuous health plan membership throughout 2015 and 2016 (but could have a gap in enrollment records of ≤30 days, as administrative gaps can occur as a result of delays in membership data processing and thus are not indicative of membership interruptions/disenrollment). Individuals with any cancer or metastatic cancer diagnoses (ICD-9140–165, 170–172, 174–176, 179–199, 200–208, 238.6/ ICD-10 C00–26.9, C30.x, C37-C41.9, C43.x, C45-C45.7, C45.9, C46-C58, C60-C76.8, C7A.x, C7B,x, C80.x, C81-C85.99, C86.x, C88.x, C90-C96.9, D03.x, D45, D47.Z9,) during this same time period were excluded.

Controls were identified using the same criteria as described above except that they had no documented mental illness diagnoses during 2015 or 2016 (they could not “start” 2016, the 12-month study period, with a diagnosis nor receive one during 2016). Matching was done separately for each group (e.g., schizophrenia controls were selected and removed from the pool of controls, then BD controls, followed by MDD controls). Controls for each group were matched on age (in 4-year bands), sex and Medicare status using stratified random sampling. Matching cases to controls was 1:2 for schizophrenia diagnosis and 1:1 each for BP and MDD diagnoses. These ratios were based on what numbers were required to find an adequate number of controls for each group.

### Measures

Non-cancer chronic pain diagnoses documented on at least 2 dates in 2016 were extracted for the matched samples. The chronic pain conditions extracted included: back pain, neck pain, limb/extremity pain, arthritis, fibromyalgia/widespread muscle pain, headache, orofacial/ear/temporomandibular pain, abdominal/bowel pain, chest pain, urogenital/pelvic/menstrual pain, fractures/contusions/sprains/strains and other painful conditions [which included sickle cell disease, complex regional pain syndrome, systemic lupus erythematosus, acquired deformities (excluding spinal disorders), spinal cord injury and neuropathic pain]. The list of ICD codes used for identifying pain conditions are available online (https://github.com/MHResearchNetwork/MHRN-Central/blob/master/WP_MHRN_SMI_painOpioids.zip).

Prescription opioid medication dispensings were also extracted for the matched samples. We were specifically interested in *chronic* opioid use, defined by prescriptions dispensed that covered at least 70 days in any 90-day period or 6+ dispensings in 2016. This definition was based on prior studies conducted at one of the MHRN sites [23, 24]. The list of NDC codes used for identifying opioid medication dispensings are also available online (https://github.com/MHResearchNetwork/MHRN-Central/blob/master/WP_MHRN_SMI_painOpioids.zip).

We also examined sociodemographic (age, sex, race/ethnicity, neighborhood socioeconomic status) and clinical characteristics of the study population using data from 2016 using methods similar to prior work [25]. Overall medical comorbidity burden was calculated using the Charlson Comorbidity Index Score (CCIS). This score consists of 19 categories of comorbidity, with each category weighted based on the adjusted risk of 1-year post-discharge mortality. The overall comorbidity score reflects the cumulative increased likelihood of mortality 1 year after discharge such that higher scores are indicative of a more severe burden of comorbidity [26]. Total health care utilization (hospitalizations, ED visits and other in-person outpatient encounters) was based on summarized data from the last 6 months of 2015. This timeframe was selected so that we had a baseline measure of recent utilization history prior to the study period (which was 2016). Multiple encounters occurring on the same day were coded as a single encounter so that we were able to count utilization *days*. In order to investigate whether any site variation existed and ensure the accuracy of the data before aggregation, preliminary data comparisons across sites were conducted. This comparison found very little site variation, supporting the stability of the aggregated estimates.

### Analyses

The primary goals of our analyses were to examine whether having a diagnosis of MDD, BD or schizophrenia/schizoaffective disorder was associated with receipt of a chronic pain diagnosis and then subsequent chronic opioid prescription dispenses. For initial bivariate models, we used t-tests for continuous variables and Pearson χ2-tests for categorical data. Multivariate analyses were conducted to evaluate (1) the odds of receiving a chronic pain-related diagnosis and (2) the odds of receiving opioids, by separate mental illness diagnosis category compared with matched controls, controlling for age, sex, Medicare status, race/ethnicity, income, medical comorbidities, healthcare utilization and chronic pain diagnoses. Results of the models were reported as adjusted odds ratios (ORs) with 95% confidence intervals (CIs).

## Results

The total number of patients identified was 377,927 (248,283 cases, 129,644 controls); however, only one-third of the available MDD cases were included in the final dataset (selected randomly) because there were not a sufficient number of controls available. The sample of persons with MDD and matched controls (total *n* = 131,488) included 72% women, 86% with a neighborhood income > $40,000 per year, was 57% White, 9% Black/African-American, 22% Hispanic/Latino, and between the ages of 18 and 70 (mean: 43.5, SD: 13.8). Individuals with MDD were more likely to have higher Charlson comorbidity scores and greater healthcare utilization than matched controls without psychiatric illness; they were also more likely to have any CNCP diagnosis (62.4% compared to 39.8% of controls) and to receive chronic opioid medications (10.1% compared to 2.4% of controls; see Table 1).

**Table 1:** Patients with Major Depressive Disorder (MDD) compared to Matched Controls

Characteristic	Patients with MDD (*N* = 65,750) n(%)/Mean ± SD	Matched Controls (N = 65,738) n(%)/Mean ± SD	Test Statistic	*p*-value
Total	65,750	65,738		
Age	43.5+ 13.8	43.5+ 13.8	*t* = −0.14	0.89
Sex				
Male	18,733 (28.5%)	18,731 (28.5%)	χ^2^ = 0	0.99
Female	47,013 (71.5%)	47,005 (71.5%)		
Medicare	6329 (9.6%)	6322 (9.6%)	χ^2^ = 0	0.96
Race				
White/Caucasian	43,647 (66.3%)	31,328 (47.7%)	χ^2^ = 5891.77	< 0.0001
Black/African-American	5894 (9.0%)	5940 (9.0%)		
Asian	3850 (5.9%)	8846 (13.5%)		
Pacific Islander	540 (0.8%)	791 (1.2%)		
Native American	672 (1.0%)	462 (0.7%)		
Other	180 (0.3%)	165 (0.3%)		
Unknown	10,944 (16.6%)	18,199 (27.7%)		
Ethnicity (Hispanic)	14,134 (21.5%)	15,274 (23.2%)	χ^2^ = 57.19	< 0.0001
Neighborhood Income				
< $40,000 per year	7771(11.8%)	7648 (11.6%)	χ^2^ = 0.12	0.73
> $40,000 per year	56,796 (86.4%)	55,566 (84.5%)		
Charlson Comorbidity Index	0.56+ 1.19	0.25+ 0.74	*t* = 56.17	< 0.0001
Total healthcare utilization in last 6 mos	8.3+ 10.7	2.3+ 4.3	*t* = 132.57	< 0.0001
Pain conditions				
Any Pain	41,036 (62.4%)	26,158 (39.8%)	χ^2^ = 6731.57	< 0.0001
Back pain	13,419 (20.4%)	5944 (9.0%)	χ^2^ = 3382.42	< 0.0001
Neck pain	6877 (10.5%)	3031 (4.6%)	χ^2^ = 1613.80	< 0.0001
Limb/extremity pain, arthritis	21,239 (32.3%)	12,449 (18.9%)	χ^2^ = 3081.38	< 0.0001
Fibromyalgia/widespread muscle	4262 (6.5%)	976 (1.5%)	χ^2^ = 2146.34	< 0.0001
Headache	8359 (12.7%)	6261 (4.8%)	χ^2^ = 2580.33	< 0.0001
Orofacial/ear/temporomandibular	728 (1.1%)	409 (0.6%)	χ^2^ = 90.22	< 0.0001
Abdominal/bowel pain	9922 (15.1%)	4679 (7.1%)	χ^2^ = 2116.78	< 0.0001
Chest pain	4995 (7.6%)	2417 (3.7%)	χ^2^ = 949.73	< 0.0001
Urogenital/pelvic/menstrual pain	3222 (4.9%)	1638 (2.5%)	χ^2^ = 535.78	< 0.0001
Fractures/contusions/sprains/strains	8542 (13.0%)	4405 (6.7%)	χ^2^ = 1465.45	< 0.0001
Other painful conditions^a^	7994 (12.2%)	3253 (5.0%)	χ^2^ = 2184.48	< 0.0001
Chronic opioid use^b^	6618 (10.1%)	1553 (2.4%)	χ^2^ = 3346.72	< 0.0001

^a^Includes sickle cell disease, complex regional pain syndrome, systemic lupus erythematosus, acquired deformities (excluding spinal disorders), spinal

cord injury, lyme disease, neuropathic pain

^b^Chronic use defined by 70+ days supply in a 90-day period, receiving 6+ dispenses in a year

The sample of persons with BP and matched controls (total *n* = 76,232) included 67% women, 85% with a neighborhood income > $40,000 per year, was 60% White, 9% Black/African-American, 18% Hispanic/Latino, and between the ages of 18 and 70 (mean: 42.7, SD: 13.3). Individuals with BP were similarly more likely to have a higher Charlson comorbidity score and a greater healthcare utilization than matched controls without any psychiatric illness; they were also more likely to have any CNCP diagnosis (61.5% compared to 40.3% of controls) and receive chronic opioid medications (10.4% compared to 3.0% of controls; see Table 2).

**Table 2:** Patients with Bipolar Disorder compared to Matched Controls

Characteristic	Patients with Bipolar Disorder (*N* = 38,117) n(%)/Mean ± SD	Matched Controls (*N* = 38,115) n(%)/Mean ± SD	Test Statistic	*p*-value
Age	42.7+ 13.2	42.7+ 13.3	*t* = −0.20	0.84
Sex				
Male	12,530(32.9%)	12,530(32.9%)	χ^2^ = 0	0.99
Female	25,585 (67.1%)	25,583 (67.1%)		
Medicare	6386 (16.8%)	6383 (16.8%)	χ^2^ = 0	0.98
Race				
White/Caucasian	27,348(71.8%)	18,408 (48.3%)	χ2 = 5450.33	< 0.0001
Black/African-American	3410 (9.0%)	3594 (9.4%)		
Asian	1782 (4.7%)	4993 (13.1%)		
Pacific Islander	281 (0.7%)	453 (1.2%)		
Native American	499 (1.3%)	281 (0.7%)		
Other	94 (0.3%)	117 (0.3%)		
Unknown	4694 (12.3%)	10,262 (27.0%)		
Ethnicity (Hispanic)	5473 (14.4%)	8307 (21.8%)	χ^2^ = 711.63	< 0.0001
Neighborhood Income				
< $40,000 per year	4985 (13.1%)	4835(12.7%)	χ^2^ = 0.41	0.52
> $40,000 per year	32,544 (85.4%)	32,006 (84.0%)		
Charlson Comorbidity Index	0.55+ 1.10	0.28+ 0.81	*t* = 39.21	< 0.0001
Total healthcare utilization in last 6 mo	8.6+ 11.3	2.4+ 4.8	*t* = 99.04	< 0.0001
Pain conditions				
Any Pain	23,423(61.5%)	15,342 (40.3%)	χ^2^ = 3426.65	< 0.0001
Back pain	7756 (20.4%)	3650 (9.6%)	χ^2^ = 1737.92	< 0.0001
Neck pain	3713 (9.7%)	1805 (4.7%)	χ^2^ = 711.12	< 0.0001
Limb/extremity pain, arthritis	12,052(31.6%)	7401 (19.4%)	χ^2^ = 1492.66	< 0.0001
Fibromyalgia/widespread muscle	2384 (6.3%)	663 (1.7%)	χ^2^ = 1012.43	< 0.0001
Headache	5000 (13.1%)	1845 (4.8%)	χ^2^ = 1597.48	< 0.0001
Orofacial/ear/temporomandibular	477 (1.3%)	237 (0.6%)	χ^2^ = 81.42	< 0.0001
Abdominal/bowel pain	5777 (15.2%)	2821 (7.4%)	χ^2^ = 1145.30	< 0.0001
Chest pain	3009 (7.9%)	1348 (3.5%)	χ^2^ = 671.51	< 0.0001
Urogenital/pelvic/menstrual pain	1925 (5.1%)	959 (2.5%)	χ^2^ = 336.23	< 0.0001
Fractures/contusions/sprains/strains	5567 (14.6%)	2743(7.2%)	χ^2^ = 1076.93	< 0.0001
Other painful conditions^a^	4137 (10.9%)	2034 (5.3%)	χ^2^ = 779.68	< 0.0001
Chronic opioid use^b^	3961 (10.4%)	1156 (3.0%)	χ^2^ = 1648.10	< 0.0001

^a^Includes sickle cell disease, complex regional pain syndrome, systemic lupus erythematosus, acquired deformities (excluding spinal disorders), spinal cord injury, lyme disease, neuropathic pain

^b^Chronic use defined by 70+ days supply in a 90-day period, receiving 6+ dispenses in a year

The sample of persons with schizophrenia and matched controls (total *n* = 38,707) included 44% women, 83% with a neighborhood income > $40,000 per year, was 51% White, 13% Black/African-American, 22% Hispanic/Latino, and between the ages of 18 and 70 (mean: 42.3, SD: 13.8). Individuals with schizophrenia had lower neighborhood-level incomes, higher Charlson comorbidity scores, and greater healthcare utilization than matched controls without any psychiatric illness; they were also slightly more likely to have any CNCP diagnosis (47.2% compared to 42.0% of controls) and receive chronic opioid medications (6.5% compared to 5.0% of controls; see Table 3).

**Table 3:** Patients with Schizophrenia compared to Matched Controls

Characteristic	Patients with Schizophrenia (*N* = 12,916) n(%)/Mean ± SD	Matched Controls (*N* = 25,791) n(%)/Mean ± SD	Test Statistic	*p*-value
Age	42.3+ 13.8	42.3+ 13.9	*t* = 0.34	0.73
Sex				
Male	7250 (56.1%)	14,459 (56.1%)	χ^2^ = 0.02	0.90
Female	5666 (43.9%)	11,332 (43.9%)		
Medicare	5144 (39.8%)	10,247 (39.7%)	χ^2^ = 0.03	0.86
Race				
White/Caucasian	6889 (53.3%)	12,770 (49.5%)	χ^2^ = 1021.25	< 0.0001
Black/African-American	2476 (19.2%)	2724 (10.6%)		
Asian	1317 (10.2%)	3008 (11.7%)		
Pacific Islander	153 (1.2%)	343 (1.3%)		
Native American	138 (1.1%)	204 (0.8%)		
Other	28 (0.2%)	44 (0.2%)		
Unknown	1907 (14.8%)	6696 (26.0%)		
Ethnicity (Hispanic)	2511 (19.4%)	6045 (23.4%)	χ^2^ = 79.87	< 0.0001
Neighborhood Income				
< $40,000 per year	2404 (18.6%)	3444 (13.4%)	χ^2^ = 170.42	< 0.0001
> $40,000 per year	10,345(80.1%)	21,650 (83.9%)		
Charlson Comorbidity Index	0.63+ 1.18	0.43+ 1.07	t = −16.58	< 0.0001
Total healthcare utilization in last 6 mo	7.9+ 10.9	2.6+ 5.6	*t* = −63.60	< 0.0001
Pain conditions				
Any Pain	6092 (47.2%)	10,835(42.0%)	χ^2^ = 92.96	< 0.0001
Back pain	1855 (14.4%)	2687 (10.4%)	χ^2^ = 129.23	< 0.0001
Neck pain	754 (5.8%)	1228 (4.8%)	χ^2^ = 20.52	< 0.0001
Limb/extremity pain, arthritis	2942 (22.8%)	5312 (20.6%)	χ^2^ = 24.41	< 0.0001
Fibromyalgia/widespread muscle	386 (3.0%)	463 (1.8%)	χ^2^ = 57.13	< 0.0001
Headache	973 (7.5%)	1264 (4.9%)	χ^2^ = 109.52	< 0.0001
Orofacial/ear/temporomandibular	112 (0.9%)	150 (0.6%)	χ^2^ = 10.44	0.0012
Abdominal/bowel pain	1497 (11.6%)	1907 (7.4%)	χ^2^ = 188.93	< 0.0001
Chest pain	975 (7.6%)	1137 (4.4%)	χ^2^ = 164.51	< 0.0001
Urogenital/pelvic/menstrual pain	280 (2.2%)	442 (1.7%)	χ^2^ = 9.69	0.0018
Fractures/contusions/sprains/strains	1392 (10.8%)	1968 (7.6%)	χ^2^ = 107.50	< 0.0001
Other painful conditions^a^	1117 (8.7%)	1884 (7.3%)	χ^2^ = 21.71	< 0.0001
Chronic opioid use^b^	845 (6.5%)	1299 (5.0%)	χ^2^ = 37.29	< 0.0001

^a^Includes sickle cell disease, Complex Regional Pain Syndrome, systemic lupus erythematosus, acquired deformities (excluding spinal disorders), spinal cord injury, Lyme disease, Neuropathic pain

^b^Chronic use defined by 70+ days supply in a 90 day period, receiving 6+ dispenses in a year

Multivariable models indicated that having a MDD (OR = 1.90; 95% CI = 1.85–1.95) or BD (OR = 1.71; 95% CI = 1.66–1.77) diagnosis was associated with increased odds of receiving a comorbid chronic pain diagnosis after controlling for age, sex, race, income, medical comorbidities and healthcare utilization. By contrast, having a schizophrenia diagnosis (OR = 0.86; 95% CI = 0.82–0.90) was associated with decreased odds of receiving a chronic pain diagnosis (see Table 4).

**Table 4:** Odds of Receiving a Chronic Pain Diagnosis and Chronic Opioid Prescriptions among Individuals with Versus without Mental Illness

Mental Illness Diagnosis	Chronic Pain Diagnosis^a^	Opioid Prescription^b^
	Adjusted OR (CI)	Adjusted OR (CI)
Major Depressive Disorder	1.90 (1.85–1.95)*	2.59 (2.44–2.75)*
Bipolar Disorder	1.71 (1.66–1.77)*	2.12 (1.97–2.28)*
Schizophrenia	0.86 (0.82–0.90)*	1.00 (0.91–1.11)

^a^Models adjusted for age, sex, race, income, medical comorbidities, and healthcare utilization

^b^Models adjusted for age, sex, race, income, medical comorbidities, healthcare utilization and chronic pain diagnosis

*p < 0.001

Having a MDD (OR = 2.59; 95% CI = 2.44–2.75) or BD (OR = 2.12; 95% CI = 1.97–2.28) diagnosis was associated with increased odds of receiving chronic opioid medications, even after controlling for age, sex, race, income, medical comorbidities, healthcare utilization and having a chronic pain diagnosis; having a schizophrenia diagnosis was not associated with receiving chronic opioid medications (see Table 4).

## Discussion

The present study found that individuals with MDD and BD diagnoses were significantly *more* likely to receive CNCP-related diagnoses compared to matched controls; by contrast, individuals with schizophrenia or schizoaffective disorder were significantly *less* likely to receive CNCP-related diagnoses compared to matched controls. These findings confirm and extend those from previous studies [17, 27, 28] and suggest that the pattern of CNCP-related diagnoses may be different for individuals with MDD or BD than for individuals with schizophrenia or schizoaffective disorder. This finding is not surprising given that symptoms of MDD and BD overlap more with each other than with symptoms of schizophrenia and schizoaffective disorder [19].

Compared to the general population, individuals with schizophrenia have increased risk of experiencing multiple physical comorbidities warranting pain control [29–32] and thus it seems counterintuitive that they were less likely to receive CNCP diagnoses than controls in the present study. There are several possible explanations for the lower prevalence of CNCP diagnoses among individuals with schizophrenia. First, there is some evidence that individuals with schizophrenia have reduced sensitivity to pain compared to individuals without psychiatric illness [33–36]. Further, antipsychotics have been shown to have analgesic qualities [37]; therefore, this decreased likelihood of receiving a pain diagnosis could reflect lower levels of pain. However, results from a recent meta-analysis indicate that antipsychotic-free patients with schizophrenia also had elevated pain thresholds compared to controls [36]. An alternative explanation may be that individuals with schizophrenia are less likely to *express* pain rather than actually experiencing less pain, either because they are unable to adequately describe the physical symptoms due to social communication impairments [38] or they withhold this information because of concerns about how they will be treated by healthcare providers. For example, Kuritzky and colleagues reported that a large percentage of people (~ 40%) with schizophrenia who had pain-related complaints indicated that they never reported these complaints in order to avoid being perceived a burden to providers and/or to avoid hospitalization [17, 39]. However, another study with Veterans Health Administration patients found that patients with schizophrenia were twice more likely to report chronic pain in comparison to those without schizophrenia [19]. Therefore, given these conflicting findings, authors of recent systematic review suggest that it is likely more appropriate to state that pain experience in schizophrenia is *disturbed or distorted* rather than decreased or absent [38].

Behavioral health clinicians may be less likely to assign pain-related diagnoses for individuals with schizophrenia because many have limited training in physical symptom management [40] and are more focused on treating psychiatric than medical concerns [41–43]; primary care clinicians may be less likely to assign pain-related diagnoses because their short consultation times make it difficult to both assess mental symptoms and conduct physical assessments. Additionally, less experienced providers may be uncomfortable with serious mental illness and may avoid intensifying their interaction with a patient by asking probing questions about physical symptoms and performing a physical exam [40]. Indeed, there is ample evidence that individuals with schizophrenia are less likely than their peers without any psychiatric illnesses to receive medical procedures and treatments for a range of conditions including cancer screening and treatment [44], use of antihypertensive and lipid-lowering drugs [45] and appropriate diabetes care (including A1C and cholesterol testing, eye and feet exams, etc.) [46, 47]. Future studies are needed to better understand providers’ decision-making with respect to diagnosing and treating pain among patients with schizophrenia.

This lack of expression and/or disclosure of pain-related complaints by patients or under-diagnosis by providers may lead to the under-detection and under-treatment of CNCP among individuals with schizophrenia. This is problematic given that CNCP among individuals with mental illness is associated with worsening of psychiatric symptoms, impaired recovery/poor therapeutic response [19, 48], greater functional incapacitation [49, 50], lower quality of life [51, 52] and increased risk of suicide [53, 54]. Therefore, it is essential to systematically assess and monitor CNCP-related conditions among individuals with schizophrenia. Psychiatrists may be particularly well-suited to oversee pain management in this population and thus need adequate education and training to equip them to do so [55].

The present study also found that individuals with MDD and BD diagnoses were over two times more likely to receive chronic opioid medication prescriptions compared to matched controls. This finding is consistent with prior literature which has similarly reported that opioids are more commonly prescribed (and prescribed at higher doses) in these populations compared to those without these mental health conditions, even after controlling for a wide array of other demographic and clinical risk factors [10, 13, 15, 16]. One explanation for this is that these individuals may present with greater pain severity [56], thereby increasing the likelihood that clinicians will prescribe an opioid and at a higher dose [57]. However, the relationship between depressive symptoms and opioid use is complex and likely bidirectional in nature, as prior research indicates that chronic opioid use can increase the risk of new-onset depression [58] as well as depression recurrence [59]. Regardless of the nature of the causal relationship, there is evidence that mental illness is associated with diminished opioid analgesia [60] and, more importantly, mental illness is a known risk factor for a range of adverse opioid-related outcomes including opioid use disorder [61–65]. Therefore, individuals most at risk for developing opioid-related problems are also more likely to be prescribed opioids [11]. Healthcare providers should be especially conservative in prescribing opioids for individuals with mental illness – or avoid opioid therapy altogether for this population, consistent with the current Canadian Medical Association recommendation [66] – and instead, favor non-pharmacological alternatives [16] such as behavioral/psychosocial approaches.

The present study has several limitations. First, opioid prescription data is based on *dispensings*, and thus may not accurately represent patients’ actual medication use. Second, we categorized patients who had more than 1 mental health diagnosis hierarchically; therefore, a patient with schizophrenia could also have had depression but he/she would not have been included in the analyses on individuals with depression. Thus our findings should be interpreted accordingly – e.g., depression is associated with an increased odds of a pain diagnosis and receipt of opioid prescriptions *when not comorbid with schizophrenia*. However, consistent with diagnostic criteria [67], we applied a hierarchy with diagnosis of schizophrenia superseding a diagnosis of mood disorder and bipolar disorder superseding a diagnosis of unipolar depression. Third, study results were derived from a sample of members of integrated payer–provider systems. There is some evidence to suggest that individuals who are more economically and socially disadvantaged may be more severely ill [68]. Therefore, our largely insured sample may underrepresent the most impaired patients. Thus, caution is urged in generalizing the findings to uninsured populations. This study’s strengths include a large, geographically and racially/ethnically diverse study population, the comparison of 3 populations with serious mental illness to matched controls, and the inclusion of important statistical confounders such as healthcare utilization in multivariate models.

## Conclusions

The presence of pain significantly impacts individuals’ engagement in and adherence to their mental health treatment and is an important moderator of treatment-related outcomes with respect to both pharmacotherapy and psychotherapy [69, 70]. Therefore, the systematic assessment and treatment of pain among individuals with mental illness is critical to short- and long-term improvements in quality of life. Given the lack of evidence about efficacy of long-term opioid treatment for CNCP and risks of drug interactions and/or use disorders, specifically among individuals with serious mental illness, non-pharmacological (e.g., behavioral/psychosocial) treatments are needed for this population. Unfortunately, barriers to accessing these types of interventions exist, such as limited patient and clinician awareness, stigma, limited capacity and reimbursement issues [69]. Consequently, there have been recent calls for engaging mental health clinicians in pain treatment for this population, as they may be particularly well-suited to assess pain symptoms, incorporate pain into treatment plans and encourage self-management activities and participation in behavioral/psychosocial treatments for pain [69]. Future research is needed to evaluate the effectiveness of involving mental health clinicians in pain management.

## Data Availability

All SAS code is provided on the MHRN GitHub site: see https://github.com/MHResearchNetwork/MHRN-Central/blob/master/WP_MHRN_SMI_painOpioids.zip Individual-level data cannot not be shared because individual patients could be re-identified; aggregated and de-identified data can be requested by contacting the first author, Dr. Ashli Owen-Smith (aowensmith@gsu.edu).

## Abbreviations

BD: Bipolar disorder
CNCP: Chronic non-cancer pain
MDD: Major depressive disorder
MHRN: Mental Health Research Network
